# Promoting health-enhancing physical activity in Europe: Surveillance, policy development and implementation 2015–2018

**DOI:** 10.1016/j.healthpol.2021.05.011

**Published:** 2021-08

**Authors:** Stephen Whiting, Romeu Mendes, Sara Tribuzi Morais, Peter Gelius, Karim Abu-Omar, Lea Nash, Ivo Rakovac, João Breda

**Affiliations:** a World Health Organization Regional Office for Europe, Copenhagen, Denmark; b European Office for the Prevention and Control of Noncommunicable Diseases, World Health Organization Regional Office for Europe, Moscow, Russian Federation; c EPIUnit – Instituto de Saúde Pública, Universidade do Porto, Porto, Portugal; d Faculdade de Desporto, Universidade do Porto, Porto, Portugal; e Department of Sport Science and Sport, Friedrich–Alexander University Erlangen–Nürnberg, Erlangen, Germany

**Keywords:** Physical activity, Exercise, Sports, Health, Policy, Europe

## Abstract

In the European Union (EU), the low levels of health-enhancing physical activity (HEPA) and high levels of sedentary behaviour are a concerning public health issue common to all Member States. In 2013, the Council of the EU recognized the need for more data related to HEPA to support policymaking across the region and proposed a monitoring framework that included 23 indicators covering different themes relevant to HEPA promotion in the EU context. In 2014, the EU Physical Activity Focal Points Network was established to support the implementation of the monitoring framework and in 2015 and 2018 surveys were conducted to collect epidemiological and policy information related to HEPA for each Member State. This paper aims to provide an update on the status of HEPA policies and surveillance in the EU and describe the changes that have occurred since 2015. In 2018, all countries had implemented more than 10 indicators, 8/28 had implemented 20 or more indicators, and only one country had completed all 23 indicators. From 2015 to 2018, 19 indicators improved, one remained unchanged, and three regressed. From the country perspective, 17 improved the number of accomplished indicators, five maintained the indicators, and five worsened the number of indicators. Overall, there has been a clear increase in the number of countries implementing HEPA policies and strategies across the different sectors, although some heterogeneity between Members Sates was still observed. Implementation of regional physical activity strategies and the establishment of the EU-wide monitoring framework appears to have had an overall positive impact on HEPA policy development and implementation.

## Highlights

• Study of trends in physical activity policy development in Europe.
• National physical activity policy development has advanced.
• Increased national policy actions in the health and education sectors.
• Monitoring and surveillance has expanded but needs to be standardised.

## 1 Introduction

Physical inactivity is a global public health issue and is estimated to be responsible for around 1 in 10 deaths each year [1]. The prevalence of physical inactivity, defined as the proportion of the population not meeting the recommended minimum level of physical activity (PA) to protect health, is estimated to be around 27.5% for adults [2] and 81.0% for children and adolescents [3] globally. A conservative estimate of the cost of physical inactivity for health-care systems worldwide was 53.8 billion in 2013 [4].

In the European Union (EU), levels of engagement in PA and sports do not seem to be increasing. Results from the 2018 Eurobarometer survey found that nearly half of European adults never exercised or played sport and that these levels of participation had not changed substantially since 2013 [5]. Low levels of PA and high levels of sedentary behaviour are also concerning among children and young people in Europe [3,6].

Physical inactivity is a global challenge and, recognizing this, World Health Organization (WHO) has specified priority areas for Member States to increase population PA in the Global Action Plan for Physical Activity [7], which was launched in 2018 and followed the development of a Physical Activity Strategy for the WHO European Region in 2015 [8]. Within the EU, an expert group developed the EU Physical Activity Guidelines in 2008 [9] which led to the official adoption of the Council of EU Recommendation on promoting health-enhancing physical activity (HEPA) across sectors in 2013 [10]. In the Recommendation, the Council recognized the need for more data related to HEPA to support policymaking across the region and proposed a monitoring framework based on the EU PA guidelines that included 23 indicators covering different themes relevant to HEPA promotion in the EU context.

In 2014, WHO and the European Commission (EC) established the EU PA Focal Points Network to facilitate collaboration between Member States, support implementation of the monitoring framework, and work collaboratively to implement the underlying regional and global strategies. In 2015, WHO/EC collected epidemiological and policy information related to HEPA for each Member State through this network. This information provided an overview of national actions in each country of the EU, and in 2018 was published in a scientific journal for the first time by Breda et al. (2018) [11] enabling comparisons of national HEPA policy implementation and helping to identify areas that needed more investment at the regional and national level. Detailed country HEPA factsheets were also published to facilitate the exchange of good practices and an overview was published that summarised the main findings [12].

In 2018, the second round of data collection was conducted, and new country HEPA factsheets were published [13]. This second round of data collection enables an assessment of the trends in PA policy development across multiple sectors in the EU for the first time, as well as monitoring of the progress made toward implementing European and national strategies to promote HEPA. This paper aims to provide an update on the status of HEPA policies and surveillance in the EU and describe the changes that have occurred since 2015.

## 2 Materials and methods

### 2.1 Monitoring framework

The monitoring framework was composed of 23 indicators related to the following themes: (i) international PA recommendations and guidelines; (ii) cross-sectoral approach; (iii) sport; (iv) health; (v) education; (vi) environment, urban planning, and public safety; (vii) working environment; (viii) senior citizens; (ix) indicators evaluation; and (x) public awareness (Table 1).

**Table 1:** The 23 indicators of the HEPA monitoring framework.

Thematic areas	Indicators	
International PA recommendations and guidelines	Indicator 1	National recommendations on physical activity for health
	Indicator 2	Adults reaching the minimum WHO recommendation on physical activity for health
	Indicator 3	Children and adolescents reaching the minimum WHO recommendation on physical activity for health
Cross-sectoral approach	Indicator 4	National government coordination mechanism and leadership on HEPA promotion
	Indicator 5	Funding allocated specifically to HEPA promotion
Sport	Indicator 6	National Sport for All policy or action plan
	Indicator 7	Sport Clubs for Health Programme
	Indicator 8	Framework to support offers to increase access to exercise facilities for socially disadvantaged groups
	Indicator 9	Target groups addressed by the national HEPA policy
Health	Indicator 10	Monitoring and surveillance of physical activity
	Indicator 11	Counselling on physical activity
	Indicator 12	Training on physical activity in the curriculum of health professionals
Education	Indicator 13	Physical education in primary and secondary schools
	Indicator 14	Schemes for school-related physical activity promotion
	Indicator 15	HEPA in training of physical education teachers
	Indicator 16	Schemes promoting active travel to school
Environment, urban planning, and public safety	Indicator 17	Level of cycling and walking
	Indicator 18	European guidelines for improving infrastructure for leisure-time physical activity
Working environment	Indicator 19	Schemes to promote active travel to work
	Indicator 20	Schemes to promote physical activity at the workplace
Senior citizens	Indicator 21	Schemes for community interventions to promote physical activity in older adults
Indicators evaluation	Indicator 22	National HEPA policies that include a plan for evaluation
Public awareness	Indicator 23	National awareness raising campaign on physical activity

PA: physical activity; WHO: World Health organization; HEPA: health-enhancing physical activity.

### 2.2 Data collection and analysis

A survey was conducted in January 2018 to collect data on the indicators and produce information for country HEPA factsheets. As in 2015, the survey tool was developed based on the definitions and detailed information on the operationalisation of the indicators and data sources published in a working document of the EC [14].

Data were collected from all 28 countries of the WHO European Region that were EU Member States at the time. Contact persons in each country were sent a questionnaire at the end of January and requested to collect data from national partners and respond to the survey by the end of May 2018. A helpdesk was maintained throughout the process, and several webinars were conducted to answer queries related to the data collection. Responses to the questionnaire were reviewed and validated by the WHO Regional Office for Europe, identifying responses where further information was necessary, checking links to source documents, and following up with focal points for more information. Data analysis was completed by September 2018 so the results reflect the situation at that time.

## 3 Results

**Fig. 1:**
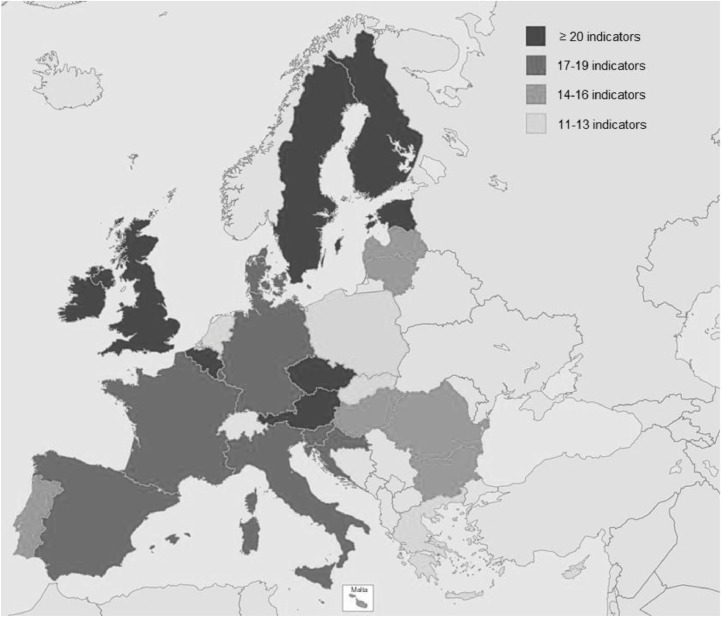
Number of implemented indicators across health-enhancing physical activity thematic areas by the European Union Member States. Map Source: EuroGeographics. Note: The designations employed and the presentation of material on this map do not imply the expression of any opinion whatsoever on the part of the European Union concerning the legal status of any country, territory, city or area or of its authorities, or concerning the delimitation of its frontiers or boundaries.

**Fig. 2:**
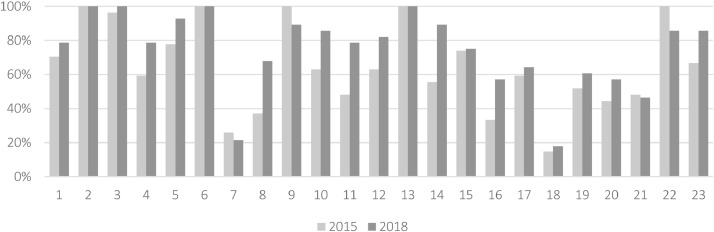
Comparison of the proportion of implementation of each health-enhancing physical activity indicator between 2015 and 2018.

All 28 European Union Member States responded to the survey on the implementation of the 23 indicators of the HEPA monitoring framework. All countries had implemented more than 10 indicators (Table 2, and Fig. 1), 8/28 had implemented 20 or more indicators, and only one country had completed all 23 indicators. In 2015, Greece did not participate in the 2015 survey and only 22/27 countries had completed more than 10 indicators, six countries had completed 20 or more indicators, and the same number of countries as in 2018 had completed all indicators. Seventeen countries increased the number of accomplished indicators, 5/28 countries Fig. 2 maintained the same number of indicators; and 5/28 achieved fewer indicators than in 2015. As for the overall results by indicator, 19/23 indicators improved, one remained unchanged, and three regressed (Table 2).

**Table 2:** Implementation of HEPA policies in line with the 23 indicators in 28 EU Member States in 2018.

	Recommendations	Cross-sectoral				Sport				Health			Education				Environment		Workplace		Senior citizens	Evaluation	Public awareness		
Indicator	1	2	3	4	5	6	7	8	9	10	11	12	13	14	15	16	17	18	19	20	21	22	23	2018 Total	2015 Total
AUT	1	1	1	1	1	1	0	1	1	1	1	1	1	1	1	1	1	1	1	1	0	0	1	20	16
BEL	1	1	1	1	1	1	0	1	1	1	1	1	1	1	1	1	1	0	1	1	1	1	1	21	21
BUL	0	1	1	1	1	1	1	0	1	1	1	1	1	1	1	0	0	0	0	0	0	1	1	15	11
CRO	1	1	1	1	1	1	0	0	1	1	1	1	1	1	0	1	0	0	1	1	0	1	1	17	9
CYP	0	1	1	1	1	1	0	0	1	0	0	1	1	1	1	0	0	0	0	0	0	0	1	11	6
CZH	1	1	1	1	1	1	1	1	1	1	1	1	1	1	1	1	1	1	1	1	1	0	0	21	16
DEN	1	1	1	0	1	1	0	1	1	1	1	1	1	1	1	1	1	0	1	1	0	1	1	19	18
DEU	1	1	1	1	1	1	0	0	1	1	1	1	1	1	1	0	1	0	1	1	1	1	1	19	21
EST	1	1	1	1	1	1	0	1	1	1	0	1	1	1	1	1	0	1	1	1	1	1	1	20	16
FIN	1	1	1	1	1	1	1	1	1	1	1	1	1	1	1	1	1	1	1	1	1	1	1	23	23
FRA	1	1	1	1	1	1	1	1	1	1	1	0	1	1	0	1	1	0	1	1	0	1	1	19	14
GRE	1	1	1	0	0	1	0	1	1	0	0	0	1	1	1	0	0	0	0	0	1	1	1	12	N/A
HUN	1	1	1	1	1	1	0	0	1	1	1	0	1	1	0	1	0	0	1	0	1	1	1	16	20
IRE	1	1	1	1	1	1	1	1	1	1	1	1	1	1	1	1	1	0	1	1	1	1	1	22	19
ITA	1	1	1	0	1	1	1	1	1	1	1	1	1	1	0	1	1	0	0	0	0	1	1	17	12
LTU	1	1	1	1	1	1	0	1	1	1	0	1	1	1	1	0	0	0	0	0	0	1	1	15	13
LUX	1	1	1	1	1	1	0	0	0	1	1	1	1	1	0	1	1	0	1	0	1	1	1	17	15
LVA	1	1	1	0	1	1	0	0	1	1	1	1	1	1	1	0	1	0	0	0	0	1	0	14	14
MAT	0	1	1	1	1	1	0	1	0	1	1	1	1	1	1	0	1	0	0	0	0	1	1	15	15
NET	1	1	1	0	1	1	0	1	1	1	0	0	1	0	0	0	1	0	1	1	0	1	0	13	14
POL	0	1	1	0	1	1	0	0	1	0	1	1	1	0	1	0	0	0	0	1	0	1	0	11	12
POR	0	1	1	1	1	1	0	1	1	1	1	1	1	1	1	0	1	0	0	0	0	1	1	16	8
ROM	0	1	1	1	1	1	0	1	1	1	0	1	1	1	1	1	0	0	0	0	1	1	1	16	10
SPA	1	1	1	1	1	1	0	1	1	1	1	1	1	1	1	0	1	0	0	1	1	1	1	19	16
SVK	1	1	1	1	0	1	0	0	0	0	1	0	1	0	0	1	1	0	1	0	0	0	1	11	9
SVN	1	1	1	1	1	1	0	1	1	1	1	1	1	1	1	0	0	0	1	1	0	1	1	18	20
SWE	1	1	1	1	1	1	0	1	1	1	1	1	1	1	1	1	1	1	1	1	1	1	1	22	13
UNK	1	1	1	1	1	1	0	1	1	1	1	1	1	1	1	1	1	0	1	1	1	1	1	21	21
Total	22	28	28	22	26	28	6	19	25	24	22	23	28	25	21	16	18	5	17	16	13	24	24		

1: implemented indicator; 0: indicator not implemented; HEPA: health-enhancing physical activity; EU: European Union; N/A: not available since Greece did not participate in the 2015 survey; Δ: changes between 2015 and 2018; Country codes are World Health Organization official; AUT: Austria; BEL: Belgium; BUL: Bulgaria; CRO: Croatia; CYP: Cyprus; CZH: Czechia; DEN: Denmark; DEU: Germany; EST: Estonia; FIN: Finland; FRA: France; GRE: Greece; HUN: Hungary; IRE: Ireland; ITA: Italy; LTU: Lithuania; LUX: Luxemburg; LVA: Latvia; MAT: Malta; NET: Netherlands; POL: Poland; POR: Portugal; ROM: Romania; SPA: Spain; SVK: Slovakia; SVN: Slovenia; SWE: Sweden; UNK: United Kingdom.

### 3.1 International PA recommendations and guidelines

National recommendations for PA for health (Indicator 1) were in place in 22 countries in 2018, an increase from 2015, when only 19 countries had national recommendations in place. In nine countries, the recommendations were solely based on WHO's recommendations for PA [15] while those in an additional 10 countries were based on both WHO's as well as other recommendations.

All countries reported data on national PA prevalence (Indicators 2 and 3) and for each country these are presented in the detailed country HEPA factsheets [13]. There were large differences in reported PA levels for adults, ranging from 11.2% to 80.4%. However, this variation should be interpreted with consideration of the instrument and cut-off used to measure if someone was physically active. Twenty-one countries used a national survey to collect data on the prevalence of PA while six used the European Health Interview Survey (EHIS) [16], five used Eurobarometer [5], and one used Global Health Observatory (GHO) estimates [17]. Nineteen countries used the WHO cut-off of a minimum of 150 min per week of moderate-intensity physical activity to calculate the prevalence.

All countries reported PA prevalence data for children and adolescents (Indicator 3). As with adults, there was great variation in prevalence reported, and again, different sampling, methods and age-ranges were used. Some countries reported that more than one survey or instruments were used, including 17 from national surveys and 15 from the HBSC study. Most countries collected data using a questionnaire but five countries also collected objective data for children using accelerometers.

### 3.2 Cross-sectoral approach

In 2018, a total of 22 countries reported that a specific national coordinating mechanism for HEPA promotion, such as a working group, advisory body or coordinating institution, had been established (Indicator 4). This represented an improvement from 2015 when 16 countries reported such a mechanism.

The survey also determined whether countries had allocated funding specifically for HEPA promotion (Indicator 5) and from which sectors. Funding for the promotion of local sports was included, but funding for elite sports (competitive sports involving professional athletes) was excluded. There was an increase in the number of countries reporting that funding had been allocated specifically to HEPA promotion, from 21 countries in 2015 to 26 countries in 2018 (Indicator 5).

### 3.3 Sport

All 28 countries reported implementing Sport for All policies and/or action plans (Indicator 6). All countries reported at least one national policy or action plan for promoting HEPA. Overall, 153 national HEPA policies or action plans were reported, averaging about five per country across different sectors (sport; health; education; environment, urban planning, and public safety; working environment; and senior citizens). Of the total HEPA policies or action plans, most were implemented in the health (71.9%), education (44.4%) and sports (65.4%) sectors.

Six countries reported that the guidelines for Sports Clubs for Health were in use (Indicator 7), which is one less than in 2015. An additional 10 countries reported that similar programmes, guidelines or frameworks were in place, but not the specific EU guidelines on which this indicator is based.

Nineteen countries reported specific policies, programmes or cost incentives to increase access to exercise facilities for socially disadvantaged groups (Indicator 8), an increase from 10 countries in 2015. Targeted groups included those disadvantaged due to socioeconomic aspects (income, socio-economic status, education or employment), age and social determinants such as gender, ethnicity, culture or religion.

Twenty-five countries reported that they had addressed at least one group with particular need for targeted actions as part of national HEPA policies (Indicator 9). Of the 153 policies and action plans to promote HEPA reported, 79 included actions that targeted special groups. The groups most often addressed were those of low socioeconomic status (38), older adults (39) and people with disabilities (34). This indicator has regressed since 2015, when all countries reported that special population groups had been targeted by at least one national HEPA policy.

### 3.4 Health

Twenty-four countries reported that they had a national health monitoring and surveillance system that included measures of population PA (Indicator 10), an increase from 17 in 2015. Countries also reported if they had an established PA surveillance or monitoring system in other sectors including sports (15 countries) education (13 countries) and transport (18 countries).

The survey asked if a country had a programme to provide counselling on or prescription of physical activity through the health care setting (Indicator 11). A national programme or scheme to promote counselling on PA by health professionals was in place in 22 countries. Health professionals involved most often included medical doctors, nurses and allied health professionals. This increased from 13 countries with national programmes in 2015.

Training on HEPA was included in the curriculum of one or more types of health professional in 23 countries (Indicator 12). In 22 of these countries, medical doctors were provided with either optional or mandatory training on PA while 17 countries offered training to physiotherapists, 18 to nurses and 11 to other health professionals such as nutritionists, occupational therapists, kinesiologists and pharmacists. In around half of the countries that included HEPA in the curricula of health professionals, it was a mandatory component of the course.

### 3.5 Education

In both 2015 and 2018, all countries that took part in the survey reported that PE classes were provided in schools (Indicator 13). The amount of time allocated to PE varied between countries and the grade level and these details are published in the country factsheets [13]. The number of hours were mandated by governments in 21 countries while, in others, some hours were optional and decided by regional or local governments, the schools or the students/parents. Seventeen countries reported that the quality of physical education classes was monitored.

National schemes for school-related PA promotion were reported in 25 countries in 2018 (Indicator 14), an increase from 15 countries in 2015. The most commonly reported scheme was after-school programmes (20 countries) while 16 countries reported schemes for active school breaks and 13 for physically active breaks during lessons.

The inclusion of HEPA as part of the curriculum for physical education teachers (Indicator 15) was reported in 21 countries in 2018, an increase of one country from 2015. In most countries, this was a mandatory part of the curriculum.

Sixteen countries reported that a national programme to promote active travel to school (Indicator 16). This increased from nine countries in 2015.

### 3.6 Environment, urban planning, and public safety

A national travel survey was reported in 18 countries in 2018 compared to 16 in 2015 (Indicator 17). There was variation between countries in the specific variables that were measured, with some measuring minutes or kilometres per days walking or cycling, while others measured average travel time per trip.

Five countries reported that the IMPALA guidelines were being applied systematically in their country (Indicator 18). A number of related national programmes and policies to improve leisure-time or recreational physical infrastructure were reported but not considered as having met this specific indicator. In 2015, four countries reported that the IMPALA guidelines were in place while six countries reported that they had plans to implement the guidelines.

### 3.7 Working environment

Seventeen countries reported that active travel to work schemes had been implemented at the national level (Indicator 19), an increase from 14 countries in 2015. National initiatives to promote PA at the workplace were in place in 16 countries (Indicator 20) compared to 12 countries in 2015. In most countries, these were led by the Ministries of Health or Sports, often in collaboration with civil society organizations.

### 3.8 Senior citizens

Thirteen countries reported that community-based interventions to promote PA to older adults were in place nationwide in both 2015 and 2018 (Indicator 21). Two countries had reported that there were plans for such a national initiative in 2015 but these had not yet been implemented as of 2018.

### 3.9 Indicators evaluation

Of the 153 HEPA policies or action plans reported in different sectors, 79 (51.6%) contained an evaluation plan (Indicator 22). In 2015, countries reported that 116/152 (76.3%) of HEPA policies or action plans had an evaluation plan in place. Twenty-four countries reported at least one evaluation plan on at least one of their HEPA policies or action plans which represents a decrease from 2015 when all countries (27) reported at least one evaluation plan.

### 3.10 Public awareness

National strategies usually include an awareness-raising campaign on PA. Implementation of a national communication campaign to promote public awareness of PA was reported by 24 countries which represents an increase of six countries since 2015 (Indicator 23).

## 4 Discussion

This paper provides an update on the status of HEPA promotion and surveillance in 28 Member States of the European Union. For the first time, an analysis of results from the second round of HEPA data collection in 2018 and their comparison with results from the first round in 2015 provide an overview of recent developments and progress related to HEPA promotion in the EU.

We found that overall, there was an increase in the number of countries meeting most indicators which provides evidence of an advance in the region in the development of HEPA policies. The country-level results enable assessments of national and regional progress toward implementing regional and global strategies for PA and noncommunicable diseases, as well as the identification of gaps that need to be addressed. At the regional level, this study enables the identification of national level actions that can be scaled-out throughout the region, as well as underserved policy areas and populations. The policy and epidemiological information published here provide important information that can be used to guide advocacy efforts and the development of policies, strategies and interventions at all levels.

The total number of countries that responded to the survey increased from 27 in 2015 to 28 in 2018 as Greece did not respond in 2015 [11]. Overall, there has been a clear increase in the number of countries implementing HEPA policies and strategies across the different sectors which reflects global trends [18]. We found that there has been an increase in the number of countries meeting the majority of the indicators. When assessing the number of countries that met each indicator, five indicators increased the most from 2015 to 2018. These indicators included: Frameworks to support offers to increase access to exercise facilities for socially disadvantaged groups (Indicator 8); Monitoring and surveillance of physical activity (Indicator 10); Counselling on physical activity (Indicator 11); Schemes for school-related physical activity promotion (Indicator 14) and; Schemes promoting active travel to school (Indicator 16), suggesting that the most progress has made in these areas across the region. The number of countries with national coordinating mechanisms for PA (Indicator 1) and countries with dedicated funding for HEPA (Indicator 4) also showed important increases from 2015 to 2018.

The process of developing national PA recommendations can be an important first step in getting PA on the national agenda, and almost all countries in the EU now have recommendations in place, while the remainder are in the process of developing them. Many countries also have recommendations for special population groups that require specific guidance for groups such as older adults, women during pregnancy and breastfeeding, and people with chronic conditions. A recent study conducted a more in-depth analysis into the actual content of national PA recommendations [19].

Due to the need for a whole-of-system approach to promote PA [20], national mechanisms are important to ensure concerted, coherent and cost-effective measures to promote PA across the range of sectors and settings that are important to increase PA of the population. There was an important increase in the number of countries with established national coordination mechanisms in place that worked on PA (Indicator 4), most of which took the form of intersectoral committees or working groups that were led by representatives of either the Ministry of Health or Ministry of Sport, which aligns with earlier findings [21].

Specific funding for HEPA is another strong indicator of action and the prioritisation of HEPA. There was an increase in the number of countries reporting that funding was provided for the promotion of HEPA (Indicator 5) and these funds most often came from the health or sports sectors, which align with previous findings that these sectors are key drivers of PA policy [22]. These advances from 2015 to 2018 indicate that governments at the highest levels recognise the need to do more to promote PA and there is a greater understanding of the need for comprehensive, whole-of-system approaches to promoting PA [7].

The promotion of PA through the health sector showed good progress from 2015 to 2018. There was an increase in the number of countries with national programmes to provide prescription or counselling for PA through primary care, which is known to be an effective intervention to increase population PA [23]. There have also been a notable expansion in the methods reported by countries including by treating exercise as medicine and counselling and prescribing PA [24] as well as by assessing PA as a vital sign [25]. There was a small increase in the number of countries that reported the inclusion of modules on HEPA in the undergraduate or postgraduate training curriculum for health professionals, which can be effective in building capacity of medical doctors [26] and allied health professionals [27] to promote PA among their patients.

Schools are an important setting for promoting PA among children and adolescents. Recent findings from the WHO European Childhood Obesity Surveillance Initiative (COSI) found significant variation in PA and sedentary behaviours among young people between countries [6], and it is encouraging that national programmes to increase PA during school time (such as active breaks during lessons, physically active learning and active recesses), as well as to promote active travel to school and extracurricular opportunities, increased in the EU from 2015 to 2018. While this demonstrates a raised awareness of the need for policies and programmes to provide more opportunities for children and young people to be active throughout the day, the continued high levels of physical inactivity indicate that implementation and ongoing evaluation will be key.

The number of countries reporting that frameworks designed to increase access to exercise facilities for socially disadvantaged groups saw one of the largest increases from 2015 to 2018. Participation in organised exercise and sports often requires resources such as registration fees, equipment and travel, which can present a barrier for low income groups [28]. These findings are encouraging as they indicate that countries recognise the need for specific, practical measures aimed at increasing PA opportunities for populations that are more susceptible to physical inactivity.

There was limited to no progress from 2015 to 2018 on certain indicators and these areas may require further attention from policy makers and implementers. Overall, three indicators (7, 9 and 22) regressed from 2015 to 2018. While it is possible that this reflects an actual change from 2018 compared to 2015, these results may reflect the difficulties of policy monitoring over time, with accurate reporting requiring highly specific definitions to ensure the same interpretation of indicators and definitions. EU PA Focal Points are also required to collaborate with in-country networks to gather information from a wide range of sectors, so reporting may depend on access to information and networks.

For some indicators it appears that the questions were too specific to effectively capture some of the concrete actions that had been implemented by countries. For example, Indicator 7 asks if the EU guidelines on ‘Sports Clubs for Health’ [29] are in place. Some countries could not report yes for this indicator even though similar guidelines were in place and the interpretation of this question may have differed between 2015 and 2018 which could explain the decrease. The other two indicators that decreased, countries with policies with specific target groups (Indicator 9) and policies with an evaluation plan (Indicator 22), could in part be due to more stringent validation process in 2018 compared to 2015 rather than a change in countries' national PA policies that did not include special populations or an evaluation plan. In future, these indicators could benefit from more objective definitions and standardisation of the validation process between data collection rounds.

Monitoring and surveillance of PA levels are essential for tracking progress toward meeting PA targets. The number of countries with monitoring and surveillance system in the health sector that included population-based measures of PA (Indicator 10) increased from 17 to 24 between 2015 and 2018, suggesting that the importance of collecting data that provides an overview of levels and engagement of PA for different populations within each country is increasingly recognised. However, there were large differences in reported prevalence of PA between countries which reflects previous findings [2,3]. The challenges related to comparing these data between countries due to differences in the sampling methods and instruments used to produce these estimates remain as they were in 2015 [11] despite ongoing attempts to standardize systems [30]. In addition, different ‘cut-offs’ or recommended levels of PA were used between countries and these differences in the definitions and terminology used to describe PA between countries may limit the comparability of data between countries.

One of the strengths of the study is the use of a standard monitoring framework applied at two-time points to allow assessment of trends in policy development and implementation in all Member States of the EU. However, comparisons between the two data collection rounds as well as between countries must be made with caution. Between 2015 and 2018 there were some changes that may have impacted the comparability of results. A new questionnaire format was used with some changes to the wording and sub-items. In addition, there were changes to the WHO coordination team administering the survey and some of the country focal points changed from 2015 to 2018 so there may have been differences in how the data were collected, reported and analysed between the two rounds. While we believe efforts to validate responses improved from 2015 to 2018, this could be further strengthened in future data collection rounds to increase confidence in the results.

Besides the overall increase in the number of countries implementing HEPA policies and strategies across the different sectors, some heterogeneity between EU Members Sates is still observed. This heterogeneity is multifactorial, and political stability, economic growth, and social equality may be key cornerstones for the development and implementation of PA policies. From the perspective of indicator achievement across the region, non-traditional PA promotion in contexts such as the workplace, the built environment, and active travel need a paradigm shift and more action to facilitate the full integration of PA in the daily life of EU citizens.

## 5 Conclusion

Implementation of the EU PA guidelines and the establishment of the monitoring framework appears to have had an overall positive impact on HEPA policy development and implementation across the EU, although some heterogeneity between Members Sates is still observed. Ongoing support for reporting on the framework may continue to drive policy development and the implementation of good practices. Better monitoring and surveillance, through the standardization of systems and improved tools, will be important to demonstrate achievement of the overall goal: an increase in population PA across the EU.

## CrediT authors’ statement

Stephen Whiting: conceptualization, methodology, validation, formal analysis, investigation, data curation, writing – original draft, writing – review and editing, visualization, project administration, Romeu Mendes: conceptualization, methodology, validation, formal analysis, data curation, writing – review and editing, visualization, project administration, supervision, Sara Tribuzi Morais: conceptualization, software, methodology, validation, formal analysis, data curation, writing – original draft, writing – review and editing, visualization, project administration, Peter Gelius: methodology, writing – original draft, writing – review and editing, Karim Abu-Omar: methodology, writing – original draft, writing – review and editing, supervision, Ivo Rakovac: conceptualization, methodology, validation, formal analysis, investigation, writing – review and editing, visualization, project administration, Lea Nash: formal analysis, investigation, data curation, writing – original draft, writing – review and editing, João Breda: conceptualization, methodology, resources, writing – review and editing, visualization, supervision, project administration, funding acquisition

## Disclaimer

The writing group takes sole responsibility for the content of this article, and the content of this article reflects the views of the authors only. SW and JB are staff members of the WHO Regional Office for Europe and RM is a WHO consultant. The authors alone are responsible for the views expressed in this article and they do not necessarily represent the views, decisions or policies of the institutions with which they are affiliated.

## Declaration of Competing Interest

None declared.
